# Abnormal hCG levels in a patient with treated stage I seminoma: a diagnostic dilemma

**DOI:** 10.1186/1477-7819-6-68

**Published:** 2008-06-25

**Authors:** Noel J Aherne, Cormac A Small, Gerard P McVey, David A Fitzpatrick, John G Armstrong

**Affiliations:** 1 Department of Radiation Oncology, St. Luke's Hospital, Dublin, Ireland; 2 Department of Radiation Oncology, University College Dublin, Dublin, Ireland

## Abstract

**Background:**

We report the case of a patient with treated Stage Ia seminoma who was found to have an elevated beta human chorionic gonadotrophin (hCG) on routine follow – up. This instigated restaging and could have lead to commencement of chemotherapy.

**Case presentation:**

The patient was a bodybuilder, and following a negative metastatic work – up, admitted to injecting exogenous beta hCG. This was done to reduce withdrawal symptoms from androgen abuse. The patient remains well eight years post diagnosis.

**Conclusion:**

This case highlights the need for surgical oncologists to conduct vigilant screening of young male patients with a history of testicular germ cell tumours and who may indulge in steroid abuse.

## Background

Testicular cancer accounts for 1% of all male cancers [1], and while the incidence has doubled in the last twenty years, the overall 5 – year survival is in the order of 99%. Among urologists and radiation oncologists, the follow – up of patients with testicular malignancies requires meticulous screening for distant metastases and careful surveillance with tumour marker measurement. These include alpha – foetoprotein (AFP) and beta human chorionic gonadotrophin (hCG), used to supplement clinical and radiologic evaluation. This case report details only the second published case of false positive beta hCG due to exogenous hCG administration [2].

## Case presentation

A 26 year old man presented to the urology service with a left testicular swelling. Clinical examination, followed by testicular ultrasound confirmed the presence of a testicular tumour. After a negative metastatic work – up, the patient proceeded to a left inguinal orchidectomy. This was followed by prophylactic para – aortic nodal irradiation to a total dose of 25 Gray (Gy) in 17 fractions. He then had a five year period of routine surveillance with clinical examination, tumour marker evaluation and annual computed tomographic (CT) scan. After five years a routine beta hCG was measured at 28.5 mIU/mL (normal 1.0 – 5.3 mIU/mL) and this raised concern regarding recurrence of his seminoma. This instigated a complete re – staging with CT Thorax/Abdomen/Pelvis and further metastatic work – up. These were all normal.

On further consultation, the patient admitted to self – administering intramuscular Nandrolone, an anabolic steroid for the previous one year. He had recently discontinued them and was taking hCG to minimise the side effects associated with their withdrawal. He discontinued hCG injections and his beta hCG normalised. He is well with no evidence of disease three years later.

## Discussion

In early stage testicular seminoma the cure rate with orchidectomy alone is up to 99% in some series. The most common area for recurrence is in the retroperitoneal and para – aortic nodes and this, coupled with their radiosensitivity, has led to the practice of adjuvant nodal irradiation in stage I seminoma for over 50 years [3]. The standard portal in this institution is from the lower border of the T10 vertebral body to the lower body of the L5 vertebra, encompassing the spinous processes and the ipsilateral renal hilum. The Medical Research Council (MRC) randomised trial, TE10, compared para – aortic strip irradiation (PAS) only with dog – leg field irradiation (DL), i.e., inclusion of the ipsilateral iliac nodes to a dose of 30 Gy in 478 patients [4]. The relapse rates in both groups were low with only nine patients relapsing in each group at 4.5 years median follow – up. During radiation treatment, nausea and vomiting, diarrhoea and, in particular, leukopenia occurred less often in the PAS arm than in the DL arm. The later MRC trial, TE 18 [5], assigned 625 patients to either 20 Gy in 10 fractions versus 30 Gy in 15 fractions. It concluded that there were no additional relapses in those receiving 20 Gy in 10 fractions versus 30 Gy in 15 fractions. Furthermore, it concluded that there was more lethargy, leucopenia and dyspepsia in the 30 Gy group.

Human chorionic gonadotrophin (hCG) is a highly specific and sensitive germ cell tumour marker. It is detectable in the serum of up to 49% of thise with seminomas at the time of diagnosis [6]. It is secreted by both seminomas and non – seminomas and while the alpha subunit is also found in other human hormones such as luteinising hormone (LH), the beta subunit is specific. A rising hCG can often precede the development of overt clinical or radiological disease and is generally taken to reflect recurrence. While most hospital assays measure the beta subunit, this would not necessarily identify exogenous administration, as seminomas can secrete the beta subunit, the intact molecule or both. A number of other malignancies can also secrete hCG (Table 1) and a false positive result can also be caused by smoking marijuana [7]. Only one previous case of a false positive result due to hCG injection has been previously described in the literature [2].

**Table 1:** Malignancies known to secrete hCG

*Gastrointestinal*
Stomach
*Hepatobiliary*
Liver
Pancreas
*Genitourinary*
Kidney
Bladder
*Other*
Breast

The illicit use of supraphysiological doses of anabolic steroids (AS) by male athletes has been common practice since the 1950 's and they are often taken in combination regimens – a process known as ' stacking '. The use of drugs such as Nandrolone in clinical practice is at doses of 50 milligrams every 3 weeks, but can be at doses of up to 800 milligrams weekly in bodybuilding and other sports where they are abused. There are many side effects associated with their use, including hepatic dysfunction, increase in total cholesterol and resultant cardiovascular morbidity, left ventricular hypertrophy, behavioural changes, thyroid dysfunction and even an increase in the risk of developing Wilms tumour, prostate adenocarcinoma and hepatocellular carcinoma. In males, even low doses of anabolic steroids cause hypogonadotrophic hypogonadism via inhibition of the production of Luteinising hormone (LH) and Follicle stimulating hormone (FSH). This can lead to diminished sperm production, testicular atrophy and gynaecomastia. The extent of the suppression of endogenous testosterone production is dependent on the strength of steroid used and the duration of the usage. Therefore, abusers seek to increase the body's own endogenous testosterone production as quickly as possible. This is done with hCG, at doses of up to 15,000 iu every three days. As hCG increases both testosterone and oestrogen, an antioestrogen such as Tamoxifen or Clomid may be taken to avoid oestrogen excess.

## Conclusion

This case highlights the problems associated with the withholding of relevant information regarding medication by patients from their doctors. An elevated hCG is often all that is needed to institute chemotherapy. In our case, this led to a number of unnecessary and costly investigations. This unusual case teaches a salutory lesson to both urologists and oncologists and illustrates the need for full disclosure by patients with seminoma of their medical history. This could prevent unnecessary investigations by urologists, radiation oncologists and medical oncologists involved in their care.

## Competing interests

The authors declare that they have no competing interests.

## Authors' contributions

NJA and CS conceived of the idea for the manuscript and performed the literature search, NJA drafted the manuscript and redrafted it after critical evaluation from GMcV and JA, JA critiqued the manuscript and defined the contextual scope of the radiation oncology management. All authors have had the opportunity to review and approve the final draft of the manuscript prior to submission.
